# Generalizing the Enhanced-Deep-Super-Resolution Neural Network to Brain MR Images: A Retrospective Study on the Cam-CAN Dataset

**DOI:** 10.1523/ENEURO.0458-22.2023

**Published:** 2024-05-24

**Authors:** Cristiana Fiscone, Nico Curti, Mattia Ceccarelli, Daniel Remondini, Claudia Testa, Raffaele Lodi, Caterina Tonon, David Neil Manners, Gastone Castellani

**Affiliations:** ^1^Department of Biomedical and Neuromotor Sciences, University of Bologna, Bologna 40126, Italy; ^2^Department of Physics and Astronomy, University of Bologna, Bologna 40126, Italy; ^3^Department of Agricultural and Food Sciences, University of Bologna, Bologna 40127, Italy; ^4^INFN, Bologna 40127, Italy; ^5^Functional and Molecular Neuroimaging Unit, IRCCS Istituto delle Scienze Neurologiche di Bologna, Bologna 40139, Italy; ^6^Department for Life Quality Studies, University of Bologna, Rimini 47921, Italy; ^7^Department of Medical and Surgical Sciences, University of Bologna, Bologna 40138, Italy

**Keywords:** brain, deep learning, image processing, MRI, super-resolution

## Abstract

The Enhanced-Deep-Super-Resolution (EDSR) model is a state-of-the-art convolutional neural network suitable for improving image spatial resolution. It was previously trained with general-purpose pictures and then, in this work, tested on biomedical magnetic resonance (MR) images, comparing the network outcomes with traditional up-sampling techniques. We explored possible changes in the model response when different MR sequences were analyzed. T_1_w and T_2_w MR brain images of 70 human healthy subjects (F:M, 40:30) from the Cambridge Centre for Ageing and Neuroscience (Cam-CAN) repository were down-sampled and then up-sampled using EDSR model and BiCubic (BC) interpolation. Several reference metrics were used to quantitatively assess the performance of up-sampling operations (RMSE, pSNR, SSIM, and HFEN). Two-dimensional and three-dimensional reconstructions were evaluated. Different brain tissues were analyzed individually. The EDSR model was superior to BC interpolation on the selected metrics, both for two- and three- dimensional reconstructions. The reference metrics showed higher quality of EDSR over BC reconstructions for all the analyzed images, with a significant difference of all the criteria in T_1_w images and of the perception-based SSIM and HFEN in T_2_w images. The analysis per tissue highlights differences in EDSR performance related to the gray-level values, showing a relative lack of outperformance in reconstructing hyperintense areas. The EDSR model, trained on general-purpose images, better reconstructs MR T_1_w and T_2_w images than BC, without any retraining or fine-tuning. These results highlight the excellent generalization ability of the network and lead to possible applications on other MR measurements.

## Significance Statement

Super-resolution applications in biomedical images may help in reducing acquisition scan time and concurrently improving the quality of the exam. Neural networks have been shown to work better than traditional up-sampling techniques, even though ad hoc training experiments need to be performed for specific kind of data. In this work, we used a model previously trained with general-purpose images, and we directly applied to magnetic resonance human brain ones; we verified its ability of reconstructing new kind of images, comparing the results with traditional up-sampling techniques. Our analysis highlights the excellent generalization capabilities of the model over the images tested, without need of specific retraining, suggesting that such results might be reproduced on images from other acquisition systems.

## Introduction

Super-Resolution (SR) algorithms aim to enhance the spatial resolution of low-resolution (LR) images, improving the detection of details and fine structures in corresponding high-resolution (HR) ones (Yang et al., 2019). The up-sampling process is an ill-posed problem by definition: starting from a smaller amount of information, the algorithms aim to increase it in the most reliable way, keeping track of several sources of noise which could affect the LR images, related to hardware (e.g., acquisition sensors, acquisition time, etc.) and/or software (e.g., compression artifacts, stochastic noise, etc) (Umer et al., 2020). Therefore, multiple putative associations between LR and HR exist, and the efficiency of up-sampling algorithms must be measured according to their ability to preserve original information or recover from degradations.

Different kinds of SR models have been developed over the years. Deep learning (DL) SR techniques were proposed by Dong et al. (2014), starting with convolutional neural networks (CNNs), a class of artificial neural networks suitable for many visual imagery applications; they have been widely used in many medical imaging tasks, including classification (Eroglu et al., 2022) and tumor detection (Çinar and Yildirim, 2020). The residual neural network (ResNet) (He et al., 2016; Dong and Inoue, 2021) has then been proposed for optimization and performance improvement: it refers to a class of models in which specific links, called residual connections, are added to a CNN structure, retaining information from different residual structural blocks and helping to overcome performance degradation problems. At first Kim et al. (2016) introduced residual learning techniques in SR tasks, which are now widely used for image restoration purposes. In this work, we used the Enhanced-Deep-Super-Resolution (EDSR) model, based on those techniques.

Deep SR neural networks have been developed in computer vision and image processing research fields, and to date they have been used in multiple real-world areas, including medical imaging (Zeng et al., 2018; Gupta et al., 2020; Sui et al., 2021; Zhang et al., 2021). In this work, we will focus on the application of the EDSR model to biomedical images, precisely to brain data acquired by magnetic resonance (MR) exams.

SR algorithms have already been implemented in clinical practice in some centers. They can bring several benefits, mainly related to the possibility of improving the trade-off between speed and accuracy of the exam. A long scan time is required for high-resolution image acquisition. This fact impacts both on the cost of the exam and on patient discomfort, which can influence the appearance of movement artifacts in the reconstructed medical images, worsening the chance of lesion detection, disease diagnosis, and radiomic feature extraction. Also, in MR imaging (MRI) applications, it leads to a reduced signal-to-noise ratio and high specific absorption rate (Sui et al., 2021). Therefore, enhancing the spatial resolution in postprocessing may help to improve the medical imaging potential (Zeng et al., 2018; Gupta et al., 2020).

It is not trivial to apply DL SR models, or DL models in general, to biomedical images: purpose-built architectures need to be tailored to a specific data type; also, the lack of data may be an issue, and data augmentation techniques (Chlap et al., 2021) are often employed to avoid overfitting; finally, DL algorithms are unsuitable for universal application, meaning that they are not guaranteed to generalize on data differing from the ones used for the training for which retraining or even start-over training stages are usually required.

In this work, we propose to apply the 2D EDSR model to MR brain images of human healthy subjects from different sequences, with 2× as up-sampling factor. We use it to evaluate 2D multislice super-resolved images, and 3D MRI reconstructions obtained from them. The major novelty of this work lies in the application of EDSR on new data avoiding retraining stages: usually, data used to train and test a DL model are of the same kind, and poor generalization over different types of data is an issue in all DL applications; here, we wanted to test the inherent generalization ability of EDSR, to establish whether it was possible to reconstruct a kind of data never seen by the model during training. In case of a positive outcome, the aim was to verify if the results achieved were superior to those of the traditional bicubic up-sampling method. As a secondary aim, we propose an original analysis on the explain ability of the model, describing the EDSR functioning with respect to the image pixel intensity. The manuscript is organized as follows: in the Materials and Methods, we provide an overview of the EDSR model used in this work, a description of the dataset and of the pipeline workflow developed for the analysis; then we describe the Results obtained by our pipeline for both 2D and 3D MRI, comparing the reconstruction in performances of the EDSR model with the standard up-sampling algorithm; we conclude this work with the description of pros and cons of our method and discuss the possibility of applying it as a postprocessing technique to speed up the MR exam acquisition and improve methodological applications such as radiomic feature extraction and artificial intelligence pipelines.

## Materials and Methods

### Ethics statement

Ethical approval for the Cam-CAN study was obtained from the Cambridgeshire 2 (now East of England-Cambridge Central) Research Ethics Committee (reference: 10/H0308/50).

### EDSR-2x model

The EDSR model is a deep 2D CNN based on residual learning techniques, and it was promoted by the SNU Computer Vision Laboratory, from Seoul National University, during the example-based single-image SR NTIRE Challenge 2017, winning the contest (Agustsson and Timofte, 2017; Lim et al., 2017; Timofte et al., 2017). EDSR was trained with end-to-end DL techniques, achieving the best reconstruction performance. Currently, it is one of the CNNs with the best results (Bashir et al., 2021; Zhang et al., 2021) even for processing biomedical images, which are the focus of this work. The single-scale architecture of EDSR was optimized starting from SRResNet (Ledig et al., 2017), itself derived from the original ResNet architecture: batch normalization layers, which are usually inserted to reduce the risk of overfitting and to guarantee fast convergence, were removed, resulting in an improvement of model time convergence, flexibility, and performance. The model was trained exploiting the DIV2K dataset, which consists of 1,000 DIVerse 2K RGB general-purpose images collected from dozens of sites, with large diversity of contents (people, handmade objects, environments, flora and fauna, etc.). During the contest, no biomedical images were used during the training and test stages; in this work, we test the model over MR brain images, without retraining or fine-tuning. In the challenge, high-resolution and corresponding low-resolution images for two, three, and four down-sampling factors were provided for training (800 images), test (100 images), and validation (100 images) stages.

### Dataset description

Data were provided by the Cambridge Centre for Ageing and Neuroscience (Cam-CAN) (Shafto et al., 2014; Taylor et al., 2017). The repository, based on a large-scale collaborative research project at the University of Cambridge, contains multimodal data, including structural and functional (resting and task-based) MR images, magnetoencephalography data, and several cognitive tests of nearly 700 healthy subjects, 100 per decade from 18 to 88 years old. We considered brain images of 70 healthy subjects (10 per decade from 18 to 85 years old), maintaining the same structure of the original collection, representative of the whole dataset. Subjects’ characteristics are summarized in Table 1.

**Table 1.** Characteristics of the analyzed samples selected from Cam-CAN dataset (Shafto et al., 2014; Taylor et al., 2017)

No of participants	70
Age (mean ± SD; years)	53.3 ± 20.0
Age range (years)	18–85
Sex (F:M)	40:30
TIV (mean ± SD; mm^3^)	(1.43 ± 0.13) × 10^6^

Cam-CAN, Cambridge Centre for Ageing and Neuroscience; SD, standard deviation; TIV, total intracranial volume.

### MRI protocol

High-resolution MR brain T_1_w and T_2_w images, acquired using a 3 T Siemens Magnetom Trio, were selected. The MR scan parameters used for the acquisition are as follows: (1) for T_1_w scans, sagittal 3D MPRAGE, TR/TE/TI = 2,250/2.99/900 ms; FA = 9°; FOV = 256 × 256 × 192 mm^3^; 1 × 1 × 1 mm^3^ spatial resolution; GRAPPA = 2; TA = 4 min 32 s; and (2) for T_2_w scans, sagittal 3D SPACE, TR/TE/TI = 2,800/408/900 ms; FOV = 256 × 256 × 192 mm^3^; 1 × 1 × 1 mm^3^ spatial resolution; GRAPPA = 2; TA = 4 min 30 s. Original images were provided in NIfTI format.

### Image processing pipeline

To generate LR maps, 2D slices from the original images were convolved with a Gaussian filter based on a 3 × 3 kernel and unit standard deviation and then two times down-sampled with BiCubic (BC) interpolation. This degradation process yields MR images with properties closed to those generated by LR MR image acquisition (Zeng et al., 2018). This pipeline is usually used in SR studies to have pairs of LR–HR images and validate the models. In fact, in an ideal situation, the same subject, perfectly still, would be scanned both with LR and HR within the same FOV, but this is unrealistic and it would be necessary to perform some registration operations which is better to avoid since interpolation methods have to be tested. Also, to make twice as many measurements would require twice of resource in terms of scan time.

The EDSR model and the BC interpolation were used to up-sample LR images. This pipeline, developed in Python 3.7.6 [libraries: OpenCV 4.5.2 (Bradski, 2000), NumPy 1.20.2, NiBabel 3.2.1], was implemented to have a gold-standard (original HR) and a comparison term (BC up-sampling, a common procedure for up-sampling) against which to assess EDSR reconstructions. After the down-sampling, images showed more pronounced ringing or Gibbs's artifacts. During preliminary stages of the work, a Gibbs-artifact removal tool (Kellner et al., 2016) was tested on LR images before the application of the two up-sampling methods, but it did not influence the outcomes. Thus, we decided to exclude this step from the pipeline.

**Figure 1.**
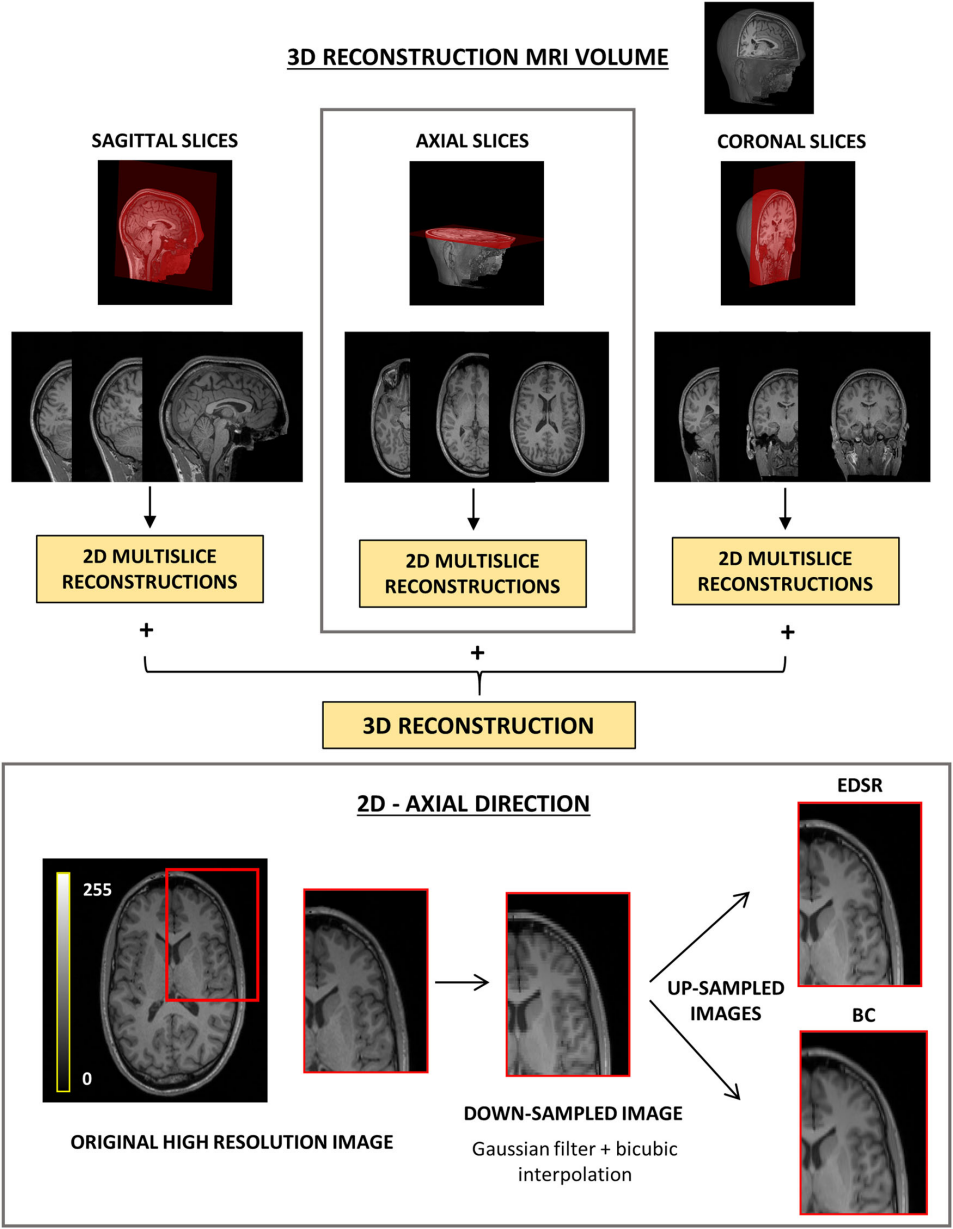
Pipeline applied to original HR T_1_w, showed in this figure as example, and T_2_w images to perform 3D reconstructions. The EDSR model is a 2D CNN, thus sagittal (192 slices), axial (256 slices), and coronal (256 slices) planes were processed separately, and three distinct 2D multislice images were achieved and then averaged to obtain the final map. The detailed pipeline for 2D SR reconstructions is illustrated in the gray box at the bottom of the panel: the original 2D images were processed with a Gaussian filter and then down-sampled with BC interpolation. The obtained LR images were up-sampled using the EDSR model and BC interpolation. SR, super-resolution; EDSR, enhanced deep super-resolution; BC, BiCubic; MRI, magnetic resonance imaging.

**Figure 2.**
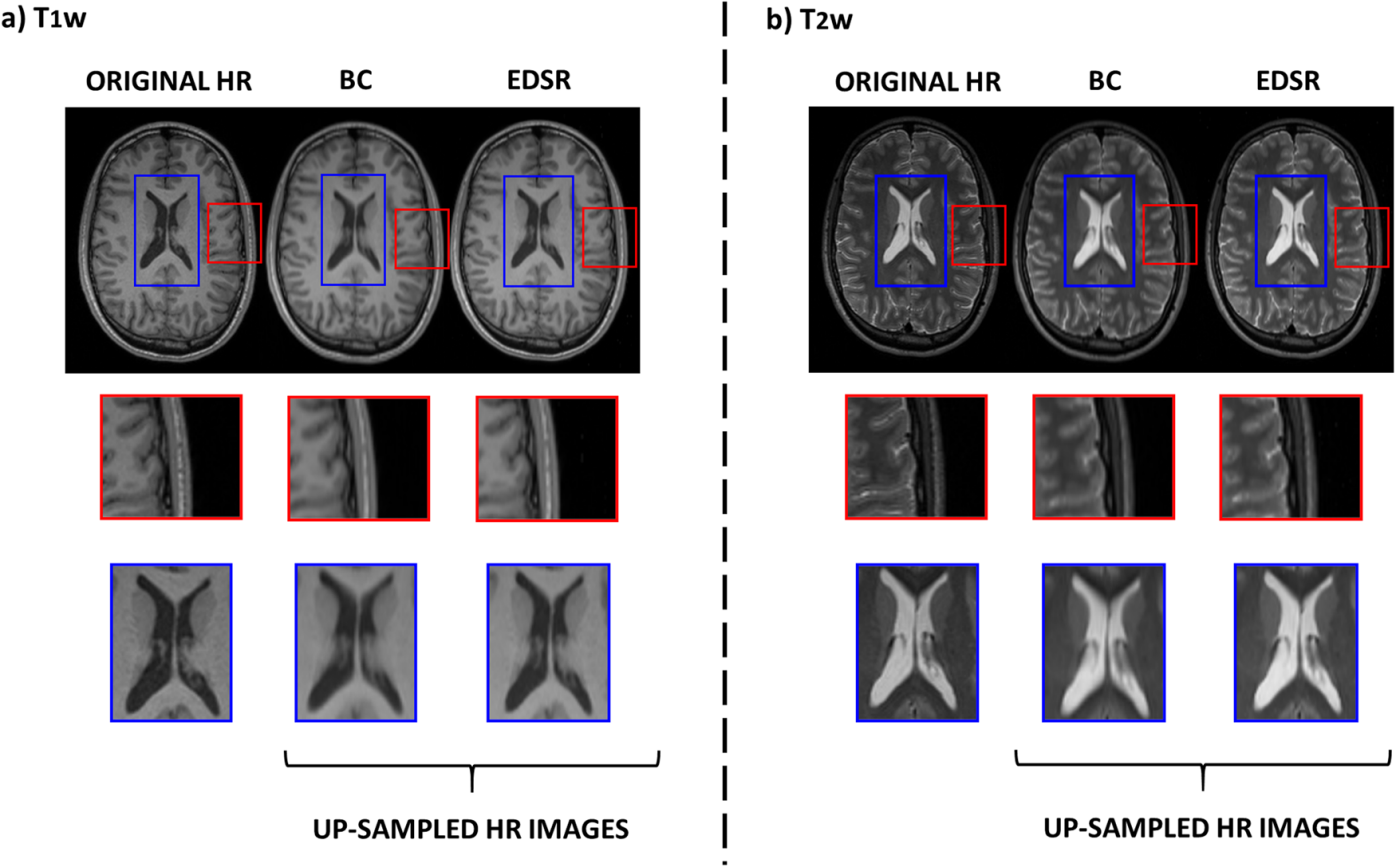
Original HR, BC, and EDSR T_1_w (panel ***a***) and T_2_w (panel ***b***) images of a representative individual (24 years/M healthy subject) from the Cam-CAN database (red boxes, zoom-in on brain cortex; blue boxes, zoom-in on brain ventricles; HR, high resolution; EDSR, enhanced deep super-resolution; BC, BiCubic).

EDSR processes 2D images, so we analyzed individually the data extracted from the three principal orthogonal planes (sagittal, axial, and coronal), yielding respectively 192, 256, and 256 processed slices. The result was a volume composed of 2D super-resolved slices for each direction. We refer to those volumes as 2D multislice reconstructions. Image dimensions for each direction are shown in Table 2. The 3D reconstructions were built up by averaging 2D multislice reconstructions from the three planes. The pipeline workflow is illustrated in Figure 1, and it was applied to both T_1_w and T_2_w images. An example of EDSR and BC reconstructions is shown in Figure 2.

**Table 2.** Matrix dimension and spatial resolution of T_1_w and T_2_w original HR, LR, and up-sampled HR images, through 2D 2x-EDSR and BC interpolation

	HR original	LR	HR EDSR and BC
Sagittal—YZ	192 × 256 × 256–1 × 1 × 1 mm^3^	192 × 128 × 128–1 × 2 × 2 mm^3^	192 × 256 × 256–1 × 1 × 1 mm^3^
Axial—XY		96 × 128 × 256–2 × 2 × 1 mm^3^	
Coronal—XZ		96 × 256 × 128–2 × 1 × 2 mm^3^	

In the three cases, the not-down-sampled dimension is underlined. HR, high resolution; LR, low resolution; EDSR, enhanced deep super-resolution; BC, BiCubic.

### Image quality assessment

The images involved in the current work were acquired for radiological studies, aiming to maximize the human readability. Therefore, the human perception plays an important role for the assessment of image quality and the similarity of images to a reference standard. To model and quantify perceived quality is challenging and in the literature several measures have been proposed (Bull and Zhang, 2021). In this work we quantified the quality of EDSR and BC images as the pixel-level fidelity to the ground truth, using standard metrics. Considering N the number of pixels, G the gold-standard image (original HR), and I the up-sampled image (EDSR or BC), we measured the following:

1. Root mean square error (RMSE) (Agustsson and Timofte, 2017):
$$\mathrm{RMSE}=\sqrt{\frac{\sum_{i=1}^{N}\;{\left(G_{i}-I_{i}\right)}^{2}}{N}}=\;\sqrt{\mathrm{MSE}}.$$
2. Peak signal-to-noise ratio (pSNR) (Timofte et al., 2017):
$$\mathrm{pSNR}=10\cdot \log_{10}\frac{{\mathrm{MAX}}_{G}^{2}}{\mathrm{MSE}}.$$

The RMSE and the pSNR are standard image quality metrics for the absolute error between the obtained image and reference one. Differences in intensity values are not enough to analyze and quantify the perceptual distortion experienced by the human vision, so the following perception-based metrics have been used in this study to provide a more comprehensive analysis:

1. Structural similarity index (SSIM) (Wang et al., 2004): is one of the most promising perception-based metrics. It considers image quality degradation as perceived change in structural information. It is a full reference metric based on visible structures in the image, extracting three features (luminance l, contrast c, and structure s) and performing the measure based on those, i.e.:
$$\mathrm{SSIM}=l\;\cdot c\;\cdot s=\;\frac{2\mu_{G}\mu_{I}+\;c_{1}}{\mu_{G}^{2}+\;\mu_{I}^{2}+\;c_{1}}\;\cdot \;\frac{2\sigma_{G}\sigma_{I}+\;c_{2}}{\sigma_{G}^{2}+\;\sigma_{I}^{2}+\;c_{2}}\cdot \;\frac{\sigma_{GI\;}+\frac{c_{2}}{2}}{\sigma_{G\;}+\sigma_{I\;}+\;\frac{c_{2}}{2}},$$

where $\mu_{G}$ is the average of G, $\mu_{I}$ is the average of I, $\sigma_{G}^{2}$ is the variance of G, $\sigma_{I}^{2}$ is the variance of I, $\sigma_{GI}$ is the covariance of G and I, $c_{1}=\;(k_{1\;}L{)}^{2}$, $c_{2}=\;(k_{2\;}L{)}^{2}$, L is the dynamic range of the pixel-values, and $k_{1\;}=0.01$ and $k_{2\;}=0.03$ by default.

1. High frequency error norm (HFEN) (Sun et al., 2019): quantifies the quality of reconstruction of edges and fine features. It is defined as L_2_ norm difference between G and I, processed by a rotationally symmetric Laplacian of Gaussian (LoG) filter of size 15 × 15 and standard deviation of 1.5, which captures the high-frequency information:
$$\mathrm{HFEN}=\;|\left|\mathrm{LoG}(G)-\mathrm{LoG}(I)\right|{|}_{2}.$$

Images with low RMSE, high pSNR, high SSIM, and low HFEN indicate high quality of reconstruction. Those metrics were evaluated with Python 3.7.6 [libraries: Scikit-image 0.16.2 (Van der Walt et al., 2014), SciPy 1.5.3 (Virtanen et al., 2020), NumPy 1.20.2] for EDSR and BC reconstructed images of each subject and each sequence, using the original HR images as ground-truth, over a region from which the scalp had been excluded using BET [Brain Extraction Tool (Smith, 2002)—FSL 6.0.4]. For 2D multislice reconstructions, the metrics were evaluated over 2D slices and the average among them were considered as follows:

$${\left\langle \mu \right\rangle }_{\mathrm{plane}}=\;\frac{1}{N_{\mathrm{plane}}}\overset{N_{\mathrm{plane}}}{\underset{\;j=1}{\sum }}\;\mu_{j},$$

where plane = sagittal, axial, and coronal; *µ* = RMSE, pSNR, SSIM, and HFEN; and *N*_plane_ = 256, 192, 256, respectively. For 3D reconstructions, similarity parameters were directly evaluated over the three-dimensional matrices.

### Comparison of up-sampling methods

The image quality metrics of EDSR and BC images were compared as follows:

1. Considering the entire brain in 2D multislice reconstruction.

Sagittal, axial, and coronal planes were considered separately. Differences between the three directions for the two up-sampling methods, EDSR, and BC were evaluated.

**Figure 3.**
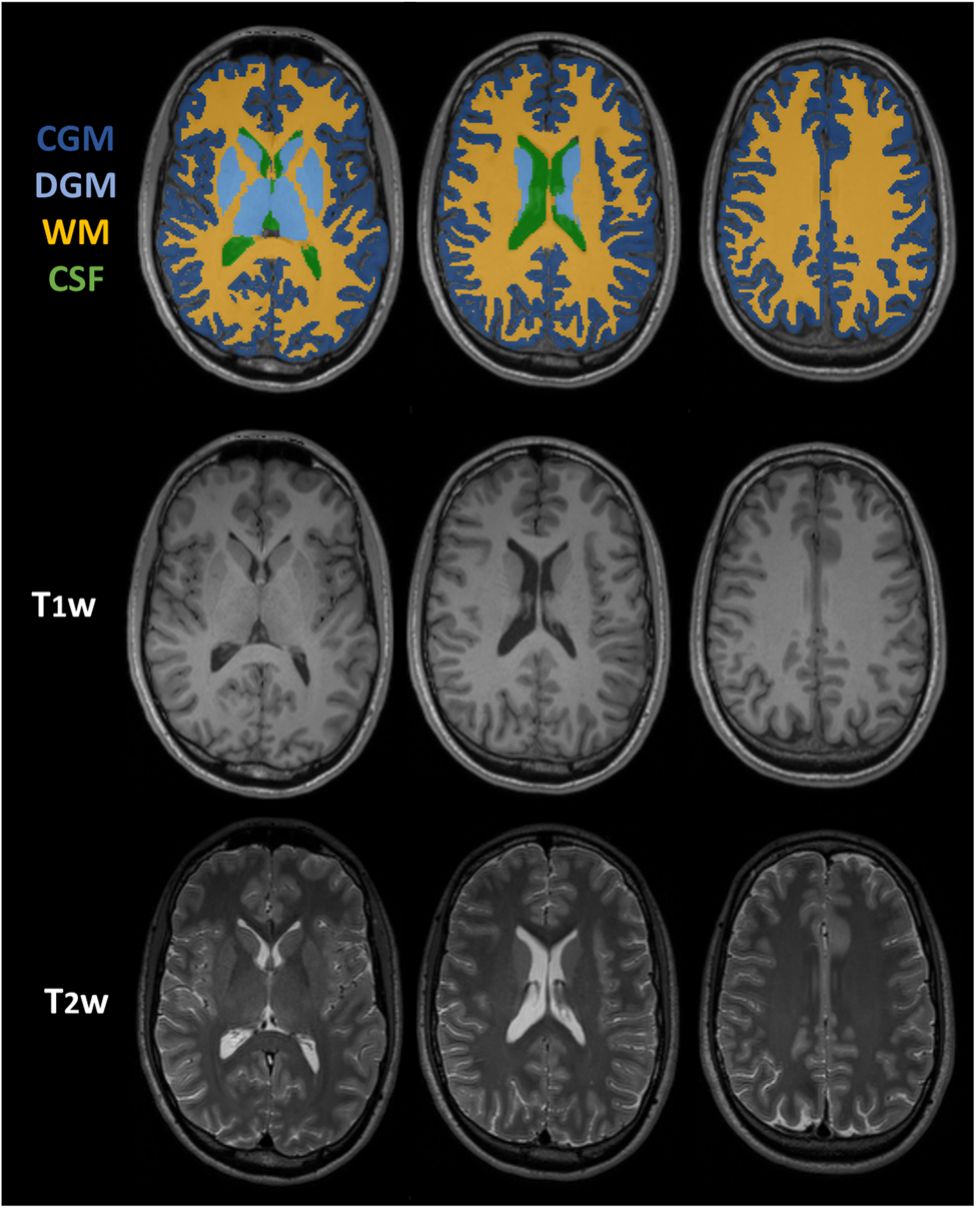
Brain tissue segmentation using 5ttgen tool from MRtrix based on a FreeSurfer parcellation image map. The considered segmented tissues are as follows: cortical cortex gray matter (CGM), deep gray matter nuclei (DGM), white matter (WM), and cerebrospinal fluid (CSF) (T_1_w and T_2_w images from 24 years/M healthy subject from the Cam-CAN database).

1. Considering the entire brain in 3D reconstructions.
2. Considering different brain tissues. The MRtrix3 (Tournier et al., 2019) tool 5ttgen, based on a FreeSurfer (Fischl, 2012) parcellation image map, was used to segment original T_1_w images into different tissues: gray matter (GM), divided into cortical cortex GM (CGM) and deep GM nuclei (DGM), white matter (WM), and cerebrospinal fluid (CSF). An example of CGM, DGM, WM, and CSF appearance in T_1_w and T_2_w images is shown in Figure 3. The up-sampled images from both T_1_w and T_2_w exams were masked using these segmentations to analyze each tissue separately. DGM and CGM were considered together to study the whole GM. 3D reconstructions were considered.
3. Considering the entire brain and inverting the gray intensity histograms. The original data included images with intensity ranging from 0 to 255. The histogram of each original image, T_1_w and T_2_w for the 70 selected subjects, was inverted within the same range. New images were obtained, in which intensities were inverted. Human perception of hyper- and hypointensity regions obviously changed after this operation. We aimed to test whether this would influence the EDSR model in performing up-sampling operation and the quantitative evaluation of its reconstructions. Therefore, after the histogram inversion, the new images were processed, and the statistical analysis was run again as described above. 3D reconstructions were considered.

We compared the distributions of RMSE, pSNR, SSIM, and HFEN obtained by EDSR and BC algorithms applied on both T_1_w and T_2_w images considering ROIs according to the above list. We used the one-sample Kolmogorov–Smirnov test to quantify the compatibility between the distributions of similarity parameters within the samples. None were normally distributed, so nonparametric comparison tests (Kruskal–Wallis and Kolmogorov–Smirnov) were selected, considering *p* values < 0.05 as significant. The effect sizes were measured by Cohen's *d* coefficients (*d *> 0.8 considered as “large”). The difference was considered significant when at least two out of three measurements showed it. Statistical analysis was carried out using Matlab R2020 (toolboxes: Statistics and Machine Learning 11.7, Image Processing 11.1).

## Results

### Entire brain: 2D reconstructions

The reference metric (RMSE, pSNR, SSIM, and HFEN) distributions among the 70-subject samples of EDSR and BC methods were evaluated as described in the previous section, using the original HR images as the gold standard for both the up-sampling methods for T_1_w and T_2_w images. Sagittal, axial, and coronal 2D slices were considered separately. Results are reported in Table 3. For each plane, each metric, and each sequence, median values [±median absolute deviation (MAD)] are shown. For all the directions, results showed significant differences in favor of the EDSR method on all the criteria for T_1_w images and on SSIM and HFEN metrics for T_2_w images. The metrics ranking among the three directions were evaluated, both for EDSR and BC methods: no orientation is superior to the others over all the measurements.

**Table 3.** Median (±MAD) of RMSE, pSNR, SSIM, and HFEN distributions among the 70-subject samples

2D—Brain	T_1_w		T_2_w	
	EDSR	BC	EDSR	BC
Sagittal YZ	RMSE ↓	32.2 ± 22.8*	77.4 ± 59.4	41.5 ± 9.1	40.0 ± 9.2
	pSNR ↑	29.1 ± 2.8*	24.7 ± 2.8	30.0 ± 1.0	30.3 ± 1.0
	SSIM ↑	0.985 ± 0.002*	0.978 ± 0.003	0.978 ± 0.002*	0.969 ± 0.002
	HFEN ↓	0.137 ± 0.040*	0.242 ± 0.045	0.174 ± 0.021*	0.226 ± 0.008
Axial XY	RMSE ↓	26.4 ± 19.7*	70.4 ± 59.3	39.6 ± 9.2	40.2 ± 8.6
	pSNR ↑	28.7 ± 3.0*	24.1 ± 3.0	29.0 ± 1.0	29.1 ± 1.0
	SSIM ↑	0.987 ± 0.002*	0.980 ± 0.003	0.978 ± 0.002*	0.969 ± 0.002
	HFEN ↓	0.138 ± 0.033*	0.237 ± 0.044	0.173 ± 0.023*	0.227 ± 0.001
Coronal XZ	RMSE ↓	38.2 ± 31.5*	97.8 ± 66.9	30.3 ± 8.2	33.7 ± 7.8
	pSNR ↑	27.7 ± 2.4*	23.8 ± 2.9	30.9 ± 1.2	30.3 ± 1.0
	SSIM ↑	0.985 ± 0.002*	0.976 ± 0.003	0.981 ± 0.002*	0.972 ± 0.002
	HFEN ↓	0.157 ± 0.042*	0.253 ± 0.053	0.151 ± 0.024*	0.230 ± 0.006

Measurements are reported for sagittal, axial, and coronal planes for T_1_w and T_2_w maps. The outperforming up-sampling method between EDSR and BC is highlighted (*) when there was a significant difference according to the considered parameters (*p* values from Kruskal–Wallis and Kolmogorov–Smirnov tests and Cohen's *d* effect size). The reference parameters were measured over the entire brain. MAD, median absolute deviation; RMSE, root mean square error; pSNR, peak signal-to-noise ratio; SSIM, structural similarity index; HEFN, high frequency error norm; EDSR, enhanced deep super-resolution; BC, BiCubic.

### Entire brain: 3D reconstructions

From the 2D multislice SR images, 3D reconstructions were obtained (Fig. 1). Results are reported in Table 4. Age, sex, and total intracranial volume (TIV) were inserted as covariates of no interest in the analysis. Results showed significant differences in favor of the EDSR method on all the metrics for T_1_w images and on SSIM and HFEN metrics for T_2_w images.

**Table 4.** Median (±MAD) of RMSE, pSNR, SSIM, and HFEN distributions among the 70-subject samples

3D—brain	T_1_w		T_2_w	
	EDSR	BC	EDSR	BC
RMSE ↓	39.1 ± 26.1*	101.9 ± 59.5	41.3 ± 9.3	46.0 ± 9.4
pSNR ↑	32.2 ± 2.3*	28.1 ± 2.8	32.0 ± 0.9	31.5 ± 0.8
SSIM ↑	0.990 ± 0.002*	0.983 ± 0.003	0.985 ± 0.001*	0.976 ± 0.002
HFEN ↓	0.144 ± 0.049*	0.244 ± 0.061	0.173 ± 0.026*	0.185 ± 0.021

Measurements are from 3D reconstructions for T_1_w and T_2_w maps. The outperforming up-sampling method between EDSR and BC is highlighted (*) when there was a significant difference according to the considered parameters (*p*-values from Kruskal–Wallis and Kolmogorov–Smirnov tests and Cohen's *d* effect size). The reference parameters were measured over the entire brain. MAD, median absolute deviation; RMSE, root mean square error; pSNR, peak signal-to-noise ratio; SSIM, structural similarity index; HEFN, high frequency error norm; EDSR, enhanced deep super-resolution; BC, BiCubic.

### Different brain tissues

We considered GM, WM, and CSF individually in 3D reconstructions. In Table 5, the results are shown, as the outcomes described in the previous section. Results showed the following: (1) in GM, significant differences in favor of the EDSR method on all the criteria for T_1_w and T_2_w images; (2) in WM, significant differences in favor of the EDSR method on all the criteria for T_2_w images, but no difference for T_1_w images; and (3) in CSF, significant differences in favor of the EDSR method on all the criteria for T_1_w images, but no difference for T_2_w images.

**Table 5.** Median (±MAD) of RMSE, pSNR, SSIM, and HFEN distributions among the 70-subject samples

3D—Brain	T_1_w		T_2_w	
	EDSR	BC	EDSR	BC
GM	RMSE ↓	11.8 ± 9.1*	14.5 ± 14.1	17.7 ± 4.7*	30.8 ± 7.2
	pSNR ↑	37.4 ± 2.6*	36.5 ± 2.6	35.6 ± 1.0*	33.3 ± 1.0
	SSIM ↑	0.998 ± 0.001*	0.998 ± 0.002	0.994 ± 0.002*	0.990 ± 0.002
	HFEN ↓	0.097 ± 0.043*	0.117 ± 0.053	0.211 ± 0.036*	0.280 ± 0.043
WM	RMSE ↓	12.4 ± 6.4	10.6 ± 7.6	6.7 ± 2.0*	10.4 ± 3.8
	pSNR ↑	37.2 ± 1.8	37.9 ± 1.9	39.9 ± 1.2*	38.0 ± 1.6
	SSIM ↑	0.998 ± 0.001	0.998 ± 0.001	0.995 ± 0.001*	0.993 ± 0.002
	HFEN ↓	0.090 ± 0.026	0.086 ± 0.028	0.195 ± 0.032*	0.240 ± 0.048
CSF	RMSE ↓	0.39 ± 0.22*	1.03 ± 0.45	1.43 ± 1.14	1.41 ± 1.05
	pSNR ↑	52.2 ± 1.7*	48.0 ± 1.6	46.6 ± 2.4	46.6 ± 2.1
	SSIM ↑	0.9998 ± 0.0002*	0.9995 ± 0.0002	0.9998 ± 0.0001	0.9998 ± 0.0001
	HFEN ↓	0.145 ± 0.029*	0.257 ± 0.040	0.172 ± 0.028	0.169 ± 0.025

Measurements are from 3D reconstructions for T_1_w and T_2_w maps. The outperforming up-sampling method between EDSR and BC is highlighted (*) when there was a significant difference according to the considered parameters (*p* values from Kruskal–Wallis and Kolmogorov–Smirnov tests and Cohen's *d* effect size). The reference parameters were measured over GM, WM, and CSF separately. MAD, median absolute deviation; RMSE, root mean square error; pSNR, peak signal-to-noise ratio; SSIM, structural similarity index; HEFN, high frequency error norm; GM, gray matter; WM, white matter; CSF, cerebrospinal fluid; EDSR, enhanced deep super-resolution; BC, BiCubic.

### Entire brain: histogram inversion

Histograms of original T_1_w and T_2_w images were inverted, and the new images were processed. Results are summarized in Table 6. There were significant differences in favor of the EDSR method on all the criteria for T_2_w images, and on the SSIM and HFEN metrics for T_1_ images.

**Table 6.** Median (±MAD) of RMSE, pSNR, SSIM, and HFEN distributions among the 70-subject samples

3D—Brain	T_1_w - INV		T_2_w - INV	
	EDSR	BC	EDSR	BC
RMSE ↓	17.1 ± 8.2	17.1 ± 9.3	44.6 ± 10.3*	67.0 ± 13.2
pSNR ↑	35.8 ± 2.0	35.8 ± 2.2	31.6 ± 0.9*	29.9 ± 0.8
SSIM ↑	0.994 ± 0.001*	0.991 ± 0.001	0.990 ± 0.001*	0.981 ± 0.001
HFEN ↓	0.048 ± 0.011*	0.052 ± 0.009	0.105 ± 0.017*	0.123 ± 0.012

Measurements are from 3D reconstructions for histogram-inverted T_1_w and T_2_w maps. The outperforming up-sampling method between EDSR and BC is highlighted (*) when there was a significant difference according to the considered parameters (*p* values from Kruskal–Wallis and Kolmogorov–Smirnov tests and Cohen's *d* effect size). The reference parameters were measured over the entire brain. MAD, median absolute deviation; RMSE, root mean square error; pSNR, peak signal-to-noise ratio; SSIM, structural similarity index; HEFN, high frequency error norm; EDSR, enhanced deep super-resolution; BC, BiCubic.

## Discussion

SR techniques have the potential to improve the trade-off between diagnostic accuracy and limited total scan time, supporting faster screening and follow-up exams. Lower resolution images can be acquired rapidly and then have their spatial resolution enhanced at the postprocessing stage. The 2D CNN EDSR model was introduced in Lim et al. (2017), and it is still one of the state-of-the-art SR DL algorithms: in a recent review of single-image SR methods (Bashir et al., 2021), EDSR was among the top 5 best performing out of a total of 19 models. It has recently been used in biomedical applications (Huang et al., 2021), and we decided to adopt it for the current study. We tested the model, pretrained on natural images, on MR scans, aiming to test its generalization ability. To validate the model, we needed a pixel-wise reference map; in an ideal situation, pairs of perfectly registered LR and HR images would be acquired during the same exam, but actually further interpolation operations would be needed to register the two images within each other, and also the required scan time would increase. Thus, following the literature (Zhang et al., 2021), we used BC interpolation, together with the application of a Gaussian filter, to simulate LR image acquisition. The results, from 2D and 3D reconstructions, were compared with BC up-sampling, chosen as comparison since it is commonly used and implemented in most image visualization software packages.

In Figure 2, an example of EDSR and BC T_1_w and T_2_w up-sampled images is shown: a qualitative assessment highlights that the model better reproduces the original HR images in both sequences, restoring high spatial frequency structures, such as the cortical gyri or ventricle edges, in more detail. To quantify aspects of reconstructions relevant to human perception, a robust analysis was carried out. The discussion follows the order of presentation of the results.

1. The model was tested over 2D images, and it outperforms BC interpolation in all the slices; there is a significant difference in favor of the EDSR model for both MR modalities considering the two perception-based metrics (SSIM and HFEN), while for the other two criteria (RMSE and pSNR) there is significant difference for T_1_w but not for T_2_w images (Table 3). Several studies have shown that SSIM and HFEN are more reliable indicators of image quality degradation and that they better match the human visual system than RMSE and pSNR (Wang et al., 2004). In fact, they measure complex image features such as luminance, contrast, and high spatial frequencies, while RMSE and pSNR are based only on the absolute difference in pixel gray levels. Thus, the results from 2D super-resolved images demonstrate that the EDSR reconstructions are better overall than those based on BC interpolation in T_1_w and T_2_w images.
  The comparison between the three orthogonal directions in both EDSR and BC methods shows that no orientation is superior to the others over all the measurements. Considering that T_1_w and T_2_w images were acquired with readout along sagittal direction, the EDSR model shows effectiveness in enhancing both in-plane (sagittal slices) and through-plane (axial and coronal slices) spatial resolution.
2. The MR images analyzed are 3D data; thus an overall assessment of the entire volume is preferred. Thus, we exploited the 2D CNN model to reproduce 3D images, combining reconstructions from the three principal orthogonal planes (Fig. 1). The analysis (Table 4) confirms the superior performance of EDSR over BC; age, sex, and TIV were inserted as covariates and did not show any influence on EDSR outcomes.
3. The reconstruction of the EDSR model showed some intensity differences compared with the original gold standard, evaluated using the RMSE and pSNR criteria. To go beyond using the algorithm as a “*black box*” and to attempt to explain its functioning, we proceeded to investigate the influence of the underlying tissue gray levels on model performance. The GM, WM, and CSF were considered separately since tissue contrast and intensities vary in the different MR sequences analyzed. The appearance of each tissue type in T_1_w and T_2_w images is shown in Figure 3, and from Table 5 it can be understood that:
  1. GM: EDSR outperforms bicubic interpolation on all criteria both in T_1_w and in T_2_w images.
  2. WM: EDSR outperforms bicubic interpolation on all criteria in T_2_w images, where the tissue appears hypointense, while in T_1_w images, where WM appears brighter, there is no significant difference.
  3. CSF: EDSR outperforms bicubic interpolation on all criteria in T_1_w images, where the tissue appears hypointense, while in T_2_w images, where CSF appears particularly hyperintense, there is no significant difference.
4. The outcomes of the previous point suggest that lack of outperformance of EDSR occurs in hyperintense areas. To test this hypothesis, histograms of the original images were reversed, and the analyses were repeated, yielding the opposite results (Table 6): the EDSR significantly outperformed BC interpolation as an up-sampling method on all the criteria in T_2_w images and in two (SSIM and HFEN) out of four in T_1_w images.

It is well established that DL algorithms, including EDSR, work better than traditional BC up-sampling over data from the training dataset (Dong et al., 2014). We wanted to test the response of EDSR to data with characteristics unlike those of the training set: the network weights, derived in the NTIRE Challenge 2017 with a training using variety of photographic images, were left untouched and the model was directly applied to MR brain images. The poor generalizability over different data is an issue in SR model applications: commonly train and test data are of the same thematic type, whichever is the model used (e.g., Generative Adversarial Networks). Some examples of brain MRI images are as follows: Zhang et al. (2021) used 3 T structural T_1_w images divided into train and test datasets; Sui et al. (2021) trained their model using 3 T T_1_w and T_2_w images from an online dataset and tested it on T_2_w images acquired on their 3T scanner. EDSR performed excellent reconstructions of MR brain images, superior to the BC standard method, leading to the consideration that a retraining stage was not necessary, although the kind of data used for the training were very different from the ones that we wanted to super-resolve. In fact, the high quality of EDSR reconstructions achieved in this work is not trivial, considering the transition from general-purpose images to brain MR scans. The capacity to generalize over new data is probably due to the large number of model parameters: referring to the review of Bashir et al. (2021), EDSR was the model with the highest number of parameters among those analyzed, more than one-third higher than the second ranked model. This fact does not affect though the computational cost, which remains low. Furthermore, EDSR is a 2D CNN, which positively influences computational effort and memory allocation. Such models can be used to reconstruct three-dimensional data, including brain images from MR exams: Zhang et al. (2021) obtained 3D reconstructions from two multislice super-resolution images, achieving better results than three-dimensional super-resolution technology. We decided to use an analogous method as a viable alternative to 3D CNNs.

Our SR pipeline showed good technical outcomes that, as we mentioned above, enable the acquisition of low-resolution images whose spatial resolution can be enhanced in postprocessing, yielding the clinical benefit of reducing the total scan time. Scan time is not linearly proportional to the voxel size, depending on several factors including parallel imaging; however, it is possible to make an approximate evaluation: for example, on a Siemens MAGNETOM Skyra 3 T scanner, the scan time for 1 mm-isotropic T_1_w (sagittal 3D, MPRAGE, TR/TE = 2,300/2.98 ms) is ∼ 5’ 21’; setting the spatial resolution to 2 mm isotropic and changing the other parameters as little as possible, it would be ∼ 2’ 30’’. Therefore, the proposed postprocessing operation could speed up the acquisition time by more than half, while yielding the same nominal spatial resolution and simultaneously reducing the probability of motion artifacts and other noise sources.

There are a few limiting factors to consider and further analysis to perform for the optimal usage of EDSR.

At first, data used in this work are high-quality MR images of healthy subjects, without sizeable movement artifacts, and their spatial resolution was improved from 2 × 2 to 1 × 1 mm^2^. At a millimeter–centimeter scale, image intensity in these images is dominated by the presence of myelinated axons (WM) neuronal and glial cell bodies (GM) and CSF. Within a 1 mm isotropic voxel, one tissue type typically predominates, and this pattern recurs in neighboring pixels over a centimeter scale, producing patches of fairly uniform pixel intensity, separated by fairly sharp boundaries. It is possible to speculate that these image characteristics are closed to those found in the photographs of natural scenes used to train the EDSR. Additionally, changes in signal intensity or morphology due to neurological diseases introduce additional tissue types in a variable manner at the voxel scale. Thus, the achievement of good performance with different scales and with pathological tissue is not obvious and will have to be tested.

At second, we expect that the model could be used on data based on MR sequences that yield contrasts other than T_1_w and T_2_w, but in this case its performance is likely to vary, especially in images with large hyperintense areas. We noticed that a deterioration in EDSR performance occurs with T_2_w images, as in Sui et al. (2021), in which CSF appears very bright. This may be related to the fact that in natural images like landscapes and animals, used to train the model, bright and shiny elements, such as light reflected in a mirror, are less common than the darker ones, such as shadows of objects and people. Moreover, images including larger glares or shadows components are commonly excluded by training datasets like the DIV2K, since they are interpreted as undesired components in a picture. To better understand and potentially explain the limits of generalizability shown by the model, we investigated its behavior with respect to the pixel intensity of the different tissue types finding that it was more difficult to accurately reproduce high-intensity pixels.

Finally, we tested EDSR over LR images generated from HR ones, which is a standard procedure in SR studies to have a pixel-wise reference maps to which compare the model reconstructions, avoiding registration operations. Nevertheless, since they are simulated LR images, the superior performance of the model needs to be confirmed on LR images from real MR acquisitions.

The aim of this work was to validate our proposed SR pipeline: the EDSR model, previously trained with natural images, was directly applied to MR brain images, without retraining or fine-tuning, to test its inherent generalization ability over a new kind of data. For this stage, we decided to use a dataset of healthy controls evaluating the performance of the model with reference metrics parameters. In future work, radiomic and textural features will be used to assess the reliability of our method, including images from patients’ exams. To assess the diagnostic relevance of the HR images obtained with the pipeline, quantitative analysis will need to be augmented with clinical evaluation.

## Conclusion

The application of the EDSR-2x on MR biomedical images in this work leads to promising results: without needing a tailored retraining, the model shows its ability to generalize from general-purpose images to different MR sequences of healthy subjects, achieving better performance than traditional up-sampling methods. Although the model works with two-dimensional data, the outperformance remains when three-dimensional reconstructions are considered. The data analyzed in this study include images from different MR sequences of subjects with different characteristics in terms of age, sex, and TIVs; several metrics and statistical tests were selected to assess the quality of the up-sampled images, thus the application of this model on MR brain images was satisfactorily validated. An analysis of the grayscale histogram identified a different reconstruction performance as function of pixel hyperintense areas, posing limits on the possible applications, especially in cases in which the MR techniques is prone to generate images with regions of high intensity.

The T_1_w and T_2_w images analyzed in this work were previously unseen by the model, and we can reasonably expect similar outcomes on images from the same sequences, which would appear with similar intensities and contrasts, acquired with different scanners.

## Synthesis

Reviewing Editor: Christoph Michel, Universite de Geneve Decisions are customarily a result of the Reviewing Editor and the peer reviewers coming together and discussing their recommendations until a consensus is reached. When revisions are invited, a fact-based synthesis statement explaining their decision and outlining what is needed to prepare a revision will be listed below. The following reviewer(s) agreed to reveal their identity: Giovanni Mettivier. Note: If this manuscript was transferred from JNeurosci and a decision was made to accept the manuscript without peer review, a brief statement to this effect will instead be what is listed below.

Both reviewers suggest to revise the manuscript.They have concrete suggestions where and how changes should be made. Please follow their advices when revising the manuscript.

Reviewer 1:

Comments to the Authors (Required) I reviewed the study titled "Generalizing the Enhanced-Deep-Super-Resolution neural network to brain MR images: a retrospective study on the Cam-CAN dataset" in detail. There are some missing points in the study. Authors need to address the following shortcomings. First of all, there are many studies in the literature using brain MRI images. https://doi.org/10.1016/j.mehy.2020.109684 , https://doi.org/10.1002/ima.22632 studies can be given as examples. Studies using MR images were not discussed in the study. At the end of the Introduction section, a paragraph containing the innovations and contributions of the article and a paragraph about the organization of the study should be added. Heading 2.1 should be elaborated because it is the most important heading of the article. Spelling and grammatical errors should be reviewed.

Reviewer 2:

In this paper the authors want to investigate the possibility of using an EDSR network previously trained on generic images to increase the spatial resolution in brain MRI images in order to reduce the acquisition time needed to obtain high spatial resolution MRI images.

The article is well written and clear and certainly of interest but there is a very important point that needs, in my opinion, more discussion. The data certainly show a superiority of the EDSR network compared to the BC method but this could also be expected since the LR images are obtained from the HR using a Gaussian filter and two BCs. However, the data does not show how well the EDSR algorithm provides an image useful for reducing the acquisition time. How close the diagnostic content is to the HR that would be obtained with a longer time acquisition. This, in my opinion, can only be assessed by a doctor. What is the opinion of the authors on the matter. Can they propose another method to evaluate it? I believe that this concept should be clarified in the discussions or included in the limitations of the study and also discuss how much this affects the achievement of the objective of the paper.
